# User Perception of Smart Home Surveillance Among Adults Aged 50 Years and Older: Scoping Review

**DOI:** 10.2196/48526

**Published:** 2024-02-09

**Authors:** Jessica Percy Campbell, Jacob Buchan, Charlene H Chu, Andria Bianchi, Jesse Hoey, Shehroz S Khan

**Affiliations:** 1 Political Science University of Victoria Victoria, BC Canada; 2 KITE Research Institute Toronto Rehabilitation Institute University Health Network Toronto, ON Canada; 3 Lawrence S Bloomberg Faculty of Nursing University of Toronto Toronto, ON Canada; 4 Centre for Clinical Ethics Unity Health Toronto Toronto, ON Canada; 5 David R Cheriton School of Computer Science University of Waterloo Waterloo, ON Canada; 6 Institute of Biomedical Engineering University of Toronto Toronto, ON Canada

**Keywords:** smart homes, privacy, surveillance, ambient assisted living, smart speakers, Internet of Things, sensors, sensor, smart home, perception, perceptions, elderly, older adult, older adults, review methods, review methodology, home monitoring, security, safety, ageing, ageing-in-place, integrative review, integrative reviews

## Abstract

**Background:**

Smart home technology (SHT) can be useful for aging in place or health-related purposes. However, surveillance studies have highlighted ethical issues with SHTs, including user privacy, security, and autonomy.

**Objective:**

As digital technology is most often designed for younger adults, this review summarizes perceptions of SHTs among users aged 50 years and older to explore their understanding of privacy, the purpose of data collection, risks and benefits, and safety.

**Methods:**

Through an integrative review, we explored community-dwelling adults’ (aged 50 years and older) perceptions of SHTs based on research questions under 4 nonmutually exclusive themes: privacy, the purpose of data collection, risk and benefits, and safety. We searched 1860 titles and abstracts from Ovid MEDLINE, Ovid Embase, Cochrane Database of Systematic Reviews, and Cochrane Central Register of Controlled Trials, Scopus, Web of Science Core Collection, and IEEE Xplore or IET Electronic Library, resulting in 15 included studies.

**Results:**

The 15 studies explored user perception of smart speakers, motion sensors, or home monitoring systems. A total of 13 (87%) studies discussed user privacy concerns regarding data collection and access. A total of 4 (27%) studies explored user knowledge of data collection purposes, 7 (47%) studies featured risk-related concerns such as data breaches and third-party misuse alongside benefits such as convenience, and 9 (60%) studies reported user enthusiasm about the potential for home safety.

**Conclusions:**

Due to the growing size of aging populations and advances in technological capabilities, regulators and designers should focus on user concerns by supporting higher levels of agency regarding data collection, use, and disclosure and by bolstering organizational accountability. This way, relevant privacy regulation and SHT design can better support user safety while diminishing potential risks to privacy, security, autonomy, or discriminatory outcomes.

## Introduction

Smart home technologies (SHTs) typically consist of one or more devices connected through the Internet of Things, which can transmit user data to various stakeholders [1]. Commonly used SHTs include Wi-Fi–enabled cameras, smart speakers with embedded voice assistants, or ambient assisted-living networks of sensors. SHTs are often controllable through smartphones, web platforms, or voice interaction [2]. These networked devices can be useful to the general population for a variety of reasons, but specifically for the aging population, they allow monitoring health status and enable information sharing with health care practitioners, family, or caregivers, potentially alleviating pressure on such networks [3-6]. Until recently, researchers have noted a gap in user perception studies focusing on older adults’ (aged 65 years and older) unique needs, preferences, and ethical factors in SHT adoption or decision-making [4]. Others have noted the need for further research that involves users from older age groups outside of the laboratory [2]. Overall, researchers have an active interest in better understanding user perceptions to remove the barriers to SHT adoption for aging populations.

Related studies have also focused on the pressing ethical implications of SHTs in terms of privacy, autonomy, and security [3,5-7]. Insights from surveillance studies and gerontology literature warn that such systems can limit user autonomy by flagging spontaneous behavior as “abnormal or deviant” in ways that could discourage users from deviating from daily routines where movements are continually monitored [3,5]. Others have noted the potential for exploiting vulnerable SHT users through surveillance capitalism, in which user behavioral data are commodified by commercial actors, resulting in exacerbated power imbalances [7,8]. For instance, some commercial-grade smart devices have the potential to make behavioral data available to advertisers, third parties, and insurance companies in ways that can disproportionately and negatively affect vulnerable individuals and groups [1,7,8]. Moreover, security issues with any internet-enabled technology can lead to unauthorized data access by malicious actors, exacerbating the potential for harm [9,10].

With these insights in mind, the aim of this paper is to explore the potential benefits and drawbacks of SHTs from the perspective of users aged 50 years and older. Despite the abovementioned privacy and security risks, it has been well established that SHT users are often limited in their knowledge of the purpose of SHT data collection [11,12]. On the other hand, SHTs are often seen as safety-enhancing [13]. Moreover, as mentioned in our related larger review paper on SHT users of all ages (Percy Campbell et al, unpublished data, January 2024), user perception studies frequently pertain to younger populations and such technology is more often designed for younger groups [14]. Because of the usual emphasis on younger age groups and technology, our goal is to incorporate the views of older demographics regarding the paradoxical benefits and drawbacks of SHTs. To do so, we collected user perception studies related to 4 nonmutually exclusive themes: privacy, the purpose of data collection, risk and benefits, and safety. To our knowledge, we are the first to compile research findings spanning these 4 categories, leading to unique insights that can inform private sector data protection regulation and SHT design, especially for older adults. We constructed four research questions prior to our literature search. (1) Privacy: What are SHT users’ privacy attitudes? (2) Purpose: What are SHT users’ understandings of the purpose of why and how their data are collected? (3) Risk or benefits: What do users think about the possible benefits and potential risks of harms of SHTs? (4) Safety: What are SHT users’ safety perceptions?

## Methods

### Overview

This section outlines our search strategy and the inclusion and exclusion criteria for paper selection. Research questions were crafted to examine the interdisciplinary literature on user perceptions of smart home surveillance. We used an integrative review framework to provide an established, rigorous, and comprehensive review method. An integrative approach is well suited to consolidating an expansive range of articles from varied theoretical backgrounds and empirical methods, allowing for a deeper understanding of a given phenomenon [15].

### Search Strategy

The search for peer-reviewed English studies was conducted in October 2021. The research team selected relevant keywords based on 4 research questions listed in the previous section. A health information specialist helped to further identify and refine the search keywords (Multimedia Appendix 1) and selected the following databases to find relevant journal articles using the following databases: Ovid MEDLINE, Ovid Embase, Cochrane Database of Systematic Reviews (Ovid), Cochrane Central Register of Controlled Trials (Ovid), Scopus; Web of Science Core Collection, and IEEE Xplore or IET Electronic Library (IEL). No time frame for the publication date was specified. The results were imported into Covidence reference management software to manage the screening process. The duplicate studies were removed automatically by Covidence and manually by team members.

**Figure 1:**
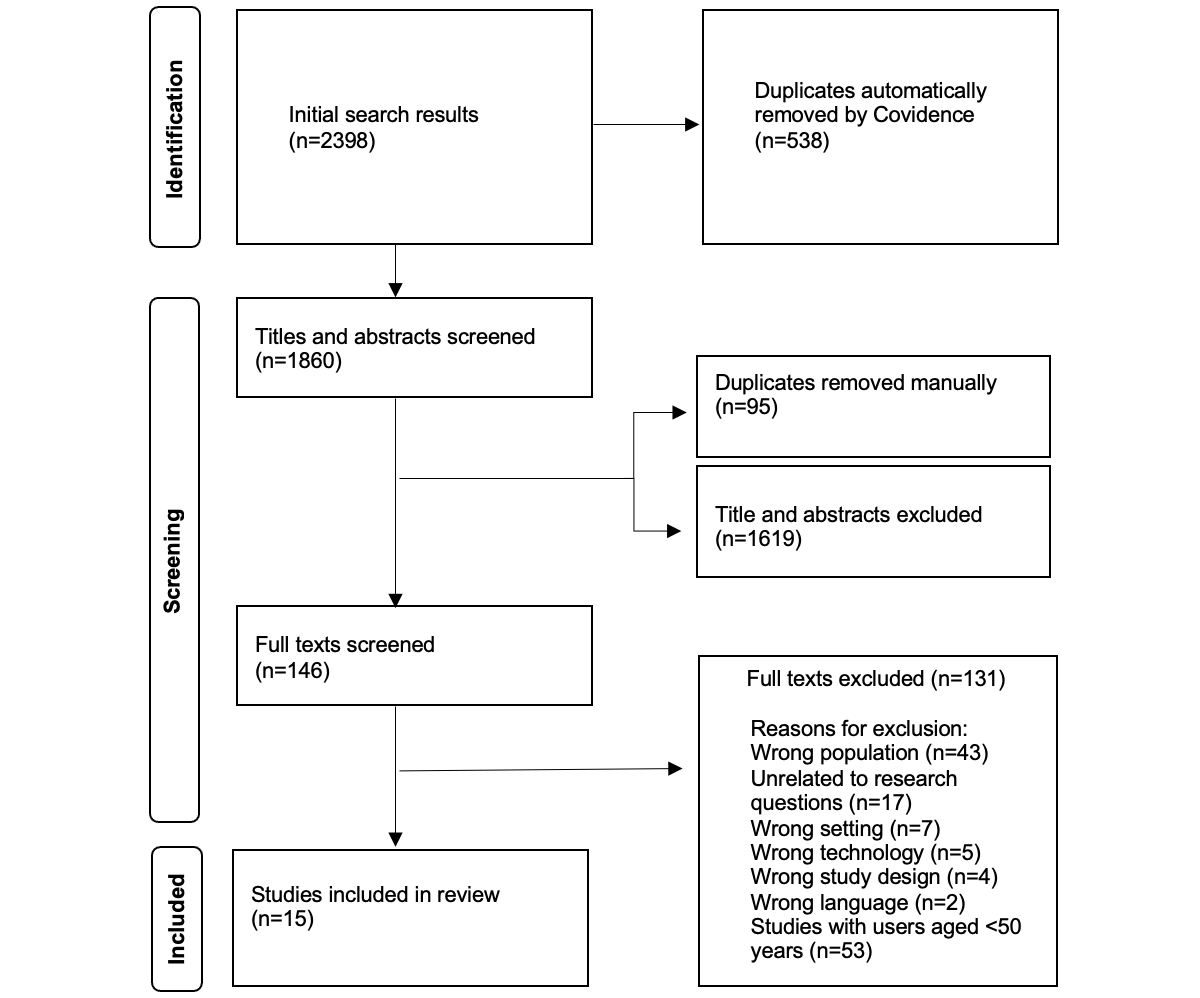
PRISMA flow diagram of extracted studies. PRISMA: Preferred Reporting Items for Systematic Reviews and Meta-Analyses.

Following duplicate removal, 4 team members were involved in the review process, which included a title or abstract screening round and a full-text review screening round. Articles were eligible for full-text review if they initially appeared to meet inclusion criteria in the title and abstract phase. Next, in the full-text review phase, each article was read in full and subsequently accepted or rejected based on inclusion and exclusion criteria. To ensure reliability and to mitigate subjective biases, article selection in each research phase required acceptance from 2 team members working independently. The rare instance of disagreement between researchers over whether to accept or reject an article was resolved through the involvement of other team members in weekly team meetings. The PRISMA (Preferred Reporting Items for Systematic Reviews and Meta-Analyses) flow diagram (Figure 1) outlines the screening processes.

### Inclusion and Exclusion Criteria

Eligible studies were those focused predominantly on smart home users’ perceptions of privacy, data collection purposes, perceived risks or benefits, and physical safety impacts of SHTs. Selected studies included SHT users aged 50 years and older, aside from 1 study where the participant age range spanned from 48 to 84 years with a mean age of 67.8 years and the aging population was the key focus [16]. The studies were included if participants were community dwelling rather than residing in care home facilities (eg, nursing homes), assisted living facilities, or hospitals. These clinical settings produce additional complexities associated with SHTs (eg, legal requirements, staff, and patient or resident consent), which were beyond the scope of this review. Qualitative, quantitative, and mixed methods empirical peer-reviewed studies that measured user perception of smart home surveillance were included. Common methods from accepted studies included questionnaires, surveys, interviews, and focus groups.

The following enumerates our exclusion criteria: (1) studies focused exclusively on wearables or smartphones due to their use outside of the home and further surveillance potential such as location tracking; (2) smart energy grid studies focused solely on cost or energy sustainability; (3) technical articles (eg, algorithm or system development) or theoretical articles; (4) system feasibility studies that were unrelated to user perception; (5) other review papers; (6) usability or acceptability studies that were unrelated to privacy, data collection purpose, risk, or safety.

### Thematic Analysis

Once the full-text screening phase had been completed, key details from each accepted study were entered into a shared Microsoft Excel sheet. The sheet was used to record the article title, author, publication year, country, method, demographic breakdown of participants (age or gender), and a short summary of key findings under the corresponding themes of privacy, the purpose of data collection, risk or benefits, and safety. The articles were classified under 1 or more themes when insights could be linked to our related research questions. These findings were then reported throughout the results section, which has been organized by theme. Summarizing articles by theme allows for patterns and contradictions to emerge from the data, ultimately facilitating analysis in the discussion section.

## Results

### Study Characteristics

Throughout the first phase, 2398 titles and abstracts were screened by our research team. The team selected 146 articles for full-text review, of which 78 were excluded based on the screening criteria mentioned in the previous section. The 68 remaining articles were selected for a larger user perception review paper on users of all age groups. Of those 68 studies, the 15 studies that focused primarily on adults aged 50 years and older were included here. Publication dates ranged from 2011 to October 2021. The results included 6 qualitative, 5 quantitative, and 4 mixed methods studies. Sample sizes ranged from 8 to 447 participants. Studies were conducted in the United States (n=4), the United Kingdom (n=3), Philippines (n=1), South Korea (n=1), Germany (n=1), Finland (n=1), the Netherlands (n=1), and 3 studies sampled participants from India, Thailand, Indonesia, and Malaysia. Participants’ mean ages ranged from 66.4 years to 86.67 years. Further demographic details are available in Table 1.

**Table 1:** Study characteristics: location, SHT^a^ type, user demographics, method, and theme.

Reference	Location	SHT type	Demographic of participants	Method	Key themes
Albina and Hernandez [17]	Philippines	Sensors, cameras	N=118 Age range: 60 to ≥81 years 34.3% female 65.7% male	Survey	Enhanced safety and privacy concerns
Arthanat et al [18]	United States	Thermostats, voice assistants, home security systems, cameras, and remote controlled lights and appliances	N=447 Age range: 65-95 years 68.8% female 31.2% male	Survey	Enhanced safety
Choi et al [2]	United States	Smart speakers, cameras, door or window sensor, and multisensor	N=37 Age range: N/A^b^ 75% female 22% male	Semistructured interview	Privacy concerns
Chung et al [19]	United States	Smart speakers (voice assistant)	N=47 Age range: N/A 49% female 51% male	Survey	Privacy concerns and risk or benefits (lack of affordability)
Ghorayeb et al [13]	United Kingdom	Nonmedical sensors: (SPHERE^c^ system: environmental and wearable and video sensors	N=13 Group 1 (users): N=6 Age range: 66-88 years 67% female 33% male Group 2 (nonuser): N=7 Age range: 67-89 years 57% female 43% male	Focus groups	Privacy concerns, safety concerns, and purpose of data collection (unaware or forgetful)
Jo et al [20]	South Korea	Environmental sensors, Bluetooth smart bands, and receivers	N=9 Age range: 68-87 years 100% female	Focus groups	Fewer privacy concerns, purpose of data collection (aware), and enhanced safety
Kirchbuchner et al [16]	Germany	Sensors	N=60 Age range: 48-84 years 70% female 30% male	Survey	Privacy concerns, risk or benefits, and enhanced safety
Leikas and Kulju [21]	Finland	Sensors	N=8 Age range: 70-85 years 88% female 12% male	Focus groups	Enhanced safety, risk or benefits (improved independent living), privacy concerns, and purpose of data collection (unaware)
O’Brien et al [22]	United States	Smart speakers (voice assistant)	N=125 Age range: N/A Sex: N/A	Thematic analysis of Amazon smart speaker reviews	Enhanced safety
Pal et al [4]	India, Thailand, Indonesia, and Malaysia	Smart homes	N=239 Age range: 55 to 75+ years 34.3% female 65.7% male	Survey	Privacy concerns and risk or benefits (security concerns)
Pal et al [23]	India, Thailand, Indonesia, and Malaysia	Smart homes	N=239 Age range: 55 to 75+ years 34.3% female 65.7% male	Survey	Privacy concerns and risk or benefits (lack of affordability)
Pal et al [24]	India, Thailand, Indonesia, and Malaysia	Smart homes	N=239 Age range: 55 to 75+ years 34.3% female 65.7% male	Survey	Privacy concerns and risk or benefits (poor design)
Psychoula et al [25]	United Kingdom	Sensors	N=41 Age range: N/A 76% female 24% male	Survey or semistructured interview	Privacy concerns (limited) and purpose of data collection (aware)
Rogerson et al [26]	United Kingdom	Sensors (light, temperature, and movement)	N=19 Age range: N/A 47% female 53% male	Semistructured interview	Privacy concerns (limited) and enhanced safety
Van Hoof et al [27]	Netherlands	Mobility monitoring, voice response, fire detection, and wandering prevention	N=12 Age range: 63-87 years 83% female 17% male	Semistructured qualitative interview	Privacy concerns (limited), enhanced safety, and risk (dependence on internet)

^a^SHT: smart home technology.

^b^N/A: not applicable.

^c^SPHERE: Sensor Platform for Healthcare in a Residential Environment

### Thematic Results

#### Overview

Table 1 shows the same papers classified under our research questions related to privacy, the purpose of data collection, risks and benefits, and safety. A total of 13 studies related to user privacy perception, 4 studies explored users’ understandings of the purpose of their data collection, 7 studies related to the benefits and risks of SHTs, while 9 studies pertained to user safety perception.

In summary, our results show that users display a diverse range of perspectives on privacy, the purpose of data collection, risks and benefits, and safety. Although privacy is often seen as an important value in need of protection, nuanced perspectives showed that users were more comfortable with certain types of devices over others, and more comfortable with certain groups of data recipients than others. Participants were sometimes unaware of SHT data collection purposes, although others expressed higher levels of understanding when their SHT functions were adequately explained to them. However, in some cases, the details of SHTs’ purpose and function were forgotten over time. Often, users lacked confidence in explaining what data their devices collected or why. Security risks, including hacking and data breaches, were often cited user concerns, while SHT convenience was frequently seen as a major benefit. Overall, despite varying levels of concern in the aforementioned areas, users were generally enthused about safety-enhancing benefits of SHTs. These safety benefits may be especially important to older adults with health concerns in particular, as SHTs can act as emergency contact tools or direct lines of support with health care providers, caregivers, and family. In what follows, the key findings are explained in further detail and separated by theme.

#### Privacy: What Are SHT Users’ Privacy Attitudes?

A total of 13 studies discussed privacy perspectives in general, with some flagging privacy as an important consideration in SHT adoption [2,17]. This section explores a range of user privacy attitudes, where environmental or motion sensors were the most accepted type of SHT [20,26] compared to smart speakers or cameras which were considered invasive [2,16], participants indicated the need to control access to their SHT data [13,21]. Many participants were skeptical about the handling of their personal information by service providers and third parties [4,23,24]. However, over time, some users forgot about the presence of sensors that collected electricity, light, temperature, and movement data [26]. In another study, most participants forgot about the presence of nonmedical sensors that collected environmental and activity data in the home [13].

Certain SHTs were generally viewed as nonthreatening, such as door and window sensors, multisensors [2], fall detection and health monitoring sensors [20], or light, temperature, and movement sensors [26]. Some participants considered sensor systems to be preferable in comparison to alternative arrangements such as institutionalization, which was generally seen as undesirable due to a lack of privacy and restricted visitation rights [27]. In early smart home models, networks of motion sensors, fall detectors, emergency voice response, and fire detection sensors were seen preferably by users, except for 1 participant who removed the technology from her home due to privacy and autonomy concerns [27]. This participant enjoyed standing in the hallway which would set off alarms, and in turn, alerted staff. However, although most participants did not feel they were being “watched or monitored” the authors also noted that “some are even not fully aware of the presence of the UAS (Unattended Autonomous Surveillance) system at home” [25].

In terms of data sharing, participants expressed mixed attitudes. In a UK study, interviews (n=41) showed older adults were open to having behavioral data collected and shared with family members or health care providers. Here, researchers noted that older adults were more open to sharing data for health care purposes than younger people [25]. One study from South Korea reported that participants (n=9) used environmental sensors for energy management and health-related sensors for fall detection and activity monitoring and reported willingness to share their health-related data with friends, family, and health care practitioners [20]. By contrast, others expressed the need to limit their data sharing to select parties. Those equipped with environmental, wearable, and video sensors in the United Kingdom preferred to share data with health care practitioners instead of family or friends (n=7) [13]. In a survey of 118 older adults (aged 60 years and older) in the Philippines, participants were concerned about assistive technology data access and sharing from environmental sensors and cameras [17].

In Germany, older adults (n=60) perceived cameras to be privacy-invasive compared to other SHTs. Here, privacy was prioritized above other potentially relevant adoption considerations, such as ease of use. Again, study participants preferred to limit data sharing, rejecting commercial service providers as legitimate data recipients [16]. Additionally, in a US study (n=37), the IP web cameras were considered more invasive than other SHTs such as smart speakers, door and window sensors, or multisensors [2]. Some participants expressed concern over smart speaker developers listening in to private conversations, while others were unperturbed [2]. Users were comfortable using smart speakers for certain purposes such as alarms, reminders, and searching for online information. However, many were hesitant to use medical SHTs that shared their health data; 1 user specifically noted their discomfort with the potential for pharmaceutical companies to profile them with targeted advertisements based on health-related data [2]. In another US survey with 47 Amazon smart speaker (Alexa) users, some participants expressed concern over their conversations being monitored, while others were indifferent [19]. The following section discusses the extent to which users understood the purpose of their SHT data collection.

#### Purpose: What Are SHT Users’ Understandings of the Purpose of Why and How Their Data Are Collected?

A total of 4 studies revealed insight into what participants understood about the purposes of their SHT data collection. In using SHTs for health care purposes, participants in Jo et al [20] were generally aware of the purposes of their sensor data collection. In this study, researchers had explained to participants what data were collected, how they were stored, and who had access to their data. However, study participants most often relied on support networks such as friends, family, or neighbors to help with their privacy decisions regarding SHTs. One issue arose, however, when participants in another study were taught about the functions of their SHTs. They eventually forgot the purposes of why those sensors were installed and, by extension, what information was being transmitted [13]. Focus group participants expressed a lack of confidence in their knowledge of whether sensors were measuring water consumption levels, humidity levels, body movement, the number of people in the room, and how alarms are triggered by artificial intelligence (AI) [13]. Similarly, focus group participants in Finland lacked confidence in their knowledge of who had access to their movement sensor data, for what purposes, and whether they had access to it themselves [21]. As noted elsewhere, SHT users often have a sense that privacy issues are present, but are unsure of “what data is collected, or how or why” [19]. Overall, apart from 1 study under this category [20], participants expressed limited understanding of the purposes of the collection of their SHT data [13,21,25]. As will be further discussed, a lack of privacy literacy around the types of surveillance SHTs contribute to is an issue with users of all age groups, younger demographics included. A lack of literacy in this area may result in difficulties in obtaining ongoing consent and informed decision-making regarding SHT use.

#### Risk and Benefits: What Do Users Think About the Possible Benefits and Potential Risks of Harm of SHTs?

A total of 7 studies explored user perceptions of SHT risks and benefits. Overall, participants identified data security threats as significant risks [16,19,21,23,24]. These perceived risks were mostly divided between disquiet over malicious data breaches, such as through hacking and misuse of personal data by smart home providers. Malicious data breaches were generally characterized by users as the unauthorized access of data by criminal parties; 1 survey (n=60) found that participants, the majority of whom had no prior experience with SHTs, were mainly concerned with criminal access to their data [16]. Similarly, the larger survey sample (n=237) in the study by Pal et al [23] showed that older adults did not trust smart home companies to securely handle their data or prevent data breaches. Specifically, the authors found that SHT users wanted their personal data to be anonymized and did not trust SHT providers to provide adequate or desired protections [23].

Alongside malicious data breaches, the misuse of personal data by SHT providers was consistently described as a risk by study participants. These concerns were mirrored in commercial contexts: focus group discussions (n=14) showed that SHT users were knowledgeable about the collection of their consumer data and were uncomfortable with their lack of agency in the use of the data [21]. Another study by Pal et al [24] (n=239) further affirmed that SHT users are uncomfortable with corporate access to their personal information. From specific medical and commercial contexts to overarching sentiments, users appear dissatisfied with the current levels of data protection offered by SHTs.

Users described additional risks beyond data collection. These included concerns over SHT dependence: semistructured interviews (n=12) demonstrated that power outages or system failures were flagged as risks by SHT users [27]. Participants also expressed concern over steep learning curves with new SHTs, as well as a potential lack of agency in selecting their own devices and controlling use of the devices [24]. Additionally, affordability was consistently identified as a risk, with concerns that SHTs would not offer benefits worth their price [4]. This finding was reiterated by Chung et al [19] where 24 of 47 (51%) surveyed users reported that affordability surpassed other risks. Overall, malfunction, affordability, and user trust represent additional risks identified by older adults using SHT.

Alongside risks, participants aged 50 years and older noted distinct positive benefits conferred by SHTs. Users were commonly enthusiastic about assistive smart home devices, including mobility monitoring, voice response, fire detection, and wandering prevention technology. Participants believed these SHTs gave them greater independence and reduced the burden on supportive family members and caregivers [21,27]. Similarly, the survey responses (n=239) in the study by Pal et al [23] indicated that users enjoyed home automation, which increased their daily convenience, especially those users who experienced or expected to experience physical or cognitive ailments. Survey participants simultaneously identified the abovementioned risks while reportedly appreciating SHTs’ value, thus creating the need to trade their reservations for SHT convenience [24]. Finally, users gained self-confidence with digital technology by mastering newly installed SHTs; however, learning to use the devices was sometimes perceived as a barrier and a deterrent to use [19]. These varied findings comprise the social benefits identified by users; however, enhanced physical safety was among the most noted. The following section explores safety perceptions in greater detail.

#### Safety: What Are SHT Users’ Safety Perceptions?

A total of 9 studies discussed the role of SHTs in safety enhancement, where participants were generally enthused about their devices’ safety features and support for aging in place [16,17,20-22,26,27]. Safety has been viewed as an important component of smart home adoption, especially for older adults with health issues [18]. Sensor users have even expressed the need to trade their privacy for increased safety through SHTs, especially for older adults living alone who experience memory problems [23].

All types of SHTs were considered useful for safety purposes, especially in an accident or emergency. In one example, stroke survivors in the United Kingdom felt safer using motion sensors in the home, as these gave them the feeling that they were being looked after [26]. In South Korea, participants found environmental and wearable sensors to be beneficial for aging in place and reported enhanced feelings of safety [20]. The ability to share their behavioral data was seen as a form of safety assurance among users [20]. Participants using ambient intelligent systems also reported enhanced feelings of safety in the home in the Netherlands, especially in the event of a fall or when feeling unwell and unable to access the phone [27]. Fall detection and other health-related safety features and burglary detection were generally well accepted [16]. Likewise, in the Philippines, assistive technology users reported enthusiasm about increased feelings of safety in the home through emergency response features [17]. Sensor Platform for Healthcare in Residential Environment users were subject to environmental, wearable, and video sensors. They were mainly concerned about the limited ability of human operators to react quickly enough in the event of an emergency [13].

Smart speakers embedded with voice assistants were also perceived to improve safety [22]. In a study examining 125 Amazon smart speaker reviews, safety features were commonly mentioned by older adults and caregivers. For example, emergency contact features such as “Ask My Buddy” were popular among reviewers [22]. In the words of one reviewer [22],

If I call out “Alexa, tell My Buddy to alert contacts,” she sends an alert via cell phone voice and text to my contact list telling them to check on me. This is great in the event of anything from a slip in the shower to any medical or emergency issue or if I feel in danger.

Throughout our collected studies, this sentiment appears to reflect the preference for the safety-enhancing features that SHTs may provide among many adults aged 50 years and older.

## Discussion

### Paradoxical Nature of SHTs

Throughout the analysis of 15 studies, study participants identified numerous benefits and drawbacks of SHTs. Overall, our findings indicated that SHT users aged 50 years and older found value in SHTs for several reasons beyond the superficial purposes of convenience or entertainment. Perceived benefits included enhanced independence levels for older adults and increased confidence levels with technology [19,21,27]. They were also widely perceived to support health and well-being through fall prevention or emergency contact features and were seen to enhance physical safety levels at home [16,17,20-22,26,27].

Conversely, participants voiced several concerns pertaining to device affordability [4,19], device reliability, criminal data breaches [16], or a lack of trust in SHT companies in securing user data [23]. If user consent over SHT data collection is to be considered meaningful, it should be ongoing, which poses an issue in cases where users expressed limited understanding of data flows and access [21]. As mentioned by homecare field professionals and related employees, this challenge can be particularly difficult for those who develop memory issues in later years [21]. This is complicated by the fact that privacy concerns sometimes fade over time and participants sometimes forget about the existence of their SHTs altogether [13,26]. Low levels of understanding around SHT data collection purposes, use, and disclosure span all age groups [28] but may be especially detrimental to older people who have SHTs installed by others for health and safety purposes.

It follows that the need for higher levels of user autonomy regarding data access is a consistent finding that requires further attention [4,16,21,23-25]. This is especially important because SHTs are often marketed in ways that promote increased autonomy for older adults. However, if SHT settings are not carefully configured and managed, they may increase independence in some ways while simultaneously diminishing it in others [28]. Data sharing and intrusive monitoring may create issues related to privacy, autonomy, or attempts at behavioral control [29]. Higher levels of user autonomy would require a strong understanding and access to controls over data monitoring and use. Otherwise, these issues can be mitigated by designers embedding tightly controlled “privacy by default” settings. The SHTs should occasionally prompt users to review and manage privacy settings and restrict data flows where unnecessary for device functionality. Finally, some older adult users may consider creating a set of guidelines and privacy preferences for caregivers to follow should memory or cognitive capacities diminish.

Despite a general unease with data sharing among third parties or service providers, one major limitation of our selected studies is the lack of detailed participant discussion on the potential for SHTs to influence insurance rates, targeted ads, or the increased difficulty in differentiating consumer data from health data. Recall that participants rejected commercial providers as data recipients [2,16]. Commercial SHTs such as smart speakers commodify user data [1,30,31], potentially inferring user health data in the process [32] and sharing such information with third parties with unknown goals or incentives. In cases where commercial-grade SHTs are used as care or safety devices for older people with health issues (eg, [19,22]), should such data still be commodified by commercial actors? SHTs can reveal mental and physical health status, mood, personality traits, and sensitive activity recognition, among other personal details [32-34]. In some cases, SHT or wearable data can also be used to influence personalized insurance rates in ways that may be disadvantage older adults with health issues [7].

Moreover, SHT developers in health care spaces have noted the difficulty in differentiating what is or should be considered medical and health data versus what is not [5]. If users are unaware of what types of data they are sharing (eg, [13,21]), to what extent is autonomous decision-making enabled or respected? When commercial-grade SHTs are used to infer health data, they may be treated as consumer data, facilitating access by public and private sector actors outside of user knowledge or meaningful consent. Many people would likely object to commercial actors gaining access to health care data from hospital settings for the purposes of third-party advertising, yet inferring user behavioral and health patterns through SHTs and wearables is possible. The discriminatory issues with targeted advertising, data brokers, and marketer classifications of different groups of people are well known [35]. Currently, studies linking the ways that SHT data contribute to targeted ads through behavioral patterns or biometric markers such as voice are in their infancy [30,33]. Additional research is needed on how SHT data from older adults are treated by SHT companies; what the subsequent targeted advertising or personalized insurance outcomes may be, either now or in the future; and whether such outcomes are discriminatory in nature.

For these reasons, the ethical implications of inferring health-related data from commercial SHT products should be considered alongside the abovementioned user privacy concerns. In short, the challenges in protecting SHT user privacy and autonomy are ongoing [3] and can be further complicated by the involvement of inferred or self-reported health-related data. As has been recommended elsewhere [2,6,14,29,36] SHT developers should prioritize design choices that better support members of all age groups through user-centric design, considering multiple stakeholders, such as older adults, nurses, and caregivers. Others have advocated for an ethical by-design (EbD) approach to implementing digital technology not only through co-design and product development but also through transdisciplinary research [29,37]. Alongside EbD choices, private sector privacy regulation could further protect users through a data justice approach that privileges human rights over commercial interests.

At a global level, the technological ability to collect and aggregate data for surveillance has outpaced regulatory mechanisms [38]. Using a data justice framework is a logical path forward to the ethical use of technology in ways that benefit both individuals and groups without further disempowering them through surveillance imperatives that do not suit their needs. Taylor’s [38] data justice framework includes three pillars: (1) visibility, (2) engagement, and (3) antidiscrimination. The first pillar, visibility, refers to the understanding that representation in certain databases can be beneficial to individuals and groups, such as in health care or welfare services. However, it also recognizes the right to privacy and the need to opt out of databases, such as those aggregated by commercial bodies [38]. As shown throughout our findings, many users indicated preferences in sharing their data with health care providers instead of family or friends [13] and preferred not to share with manufacturers, marketers, or other third parties [16]. The second pillar, digital engagement and disengagement, supports individual autonomy by encouraging personalized decisions regarding a user’s preferred level of technological engagement and control over circumstances [38]. For our purposes, the right to digital disengagement would help support older adult SHT users in situations where personalized human care is their preferred option for certain purposes or where only select SHT functions were preferred. The third pillar, the right to challenge data-driven discrimination, allows for the ability to challenge bias in algorithmic decision-making and outcomes [38]. This last pillar may be particularly important as AI capabilities continue to develop alongside rising SHT popularity. Although issues with gender and racial bias with AI platforms are well documented within the literature, digital ageism is currently understudied and is thus in need of further critical analysis [28,36]. The ability to evaluate and challenge ageist bias is an important task as consumer-grade devices become more popular among aging populations. Taken together, regulatory frameworks following Taylor’s [38] 3 pillars of data justice can be used to construct meaningful guidelines around how SHT data should be managed by private sector actors. This way, those who choose to engage with such technology in their homes can enjoy the potential health and safety benefits of SHTs while preventing or mitigating challenges to privacy, autonomy, and discrimination that can be detrimental to older age groups.

### Strengths and Limitations

To the best of our knowledge, we are the first to research SHT user perception under the 4 themes of privacy, the purpose of data collection, risk and benefits, and safety. Previous reviews have largely focused on rehabilitation or health care settings exclusively, whereas we have also incorporated user perception of commercial SHT surveillance. Our review engaged with interdisciplinary fields across the social sciences, computer sciences, engineering, legal studies, and nursing. We have also applied insights from the surveillance studies literature to findings from gerontology research. In terms of limitations, as we excluded studies that focused solely on nonusers, we may have missed potential insight into why individuals do not adopt SHTs. We also excluded studies on smartphones or wearable devices, due to their ability to be used outside the home, which may have further limited our findings. We did not include other search methods such as hand searching for references and did not reconduct the search after October 2021 both of which may have resulted in additional relevant studies. We did not conduct a quality appraisal of our included studies, resulting in another potential limitation. As many of these studies were written about users in global North countries, the extent to which these findings are representative of other regions requires further inquiry. Finally, only English language studies were reviewed, so relevant non-English papers may have been omitted.

### Conclusions

In conclusion, through our review of 15 studies, we have demonstrated a variety of perceived benefits and drawbacks from research participants over the age of 50 years. Although SHTs are seen as beneficial for safety enhancement such as emergency contact and convenience purposes, many users are also concerned about the privacy and or security risks, such as a lack of knowledge over where their data were going or a lack of control over who had access. These findings add to the growing body of literature highlighting the need for more age-inclusive technology design. This becomes especially important as commercial-grade SHTs are increasingly positioned to be used for care or health-related purposes for aging populations. In tandem with age-inclusive efforts such as EbD approaches [29], we further encourage the use and development of technology that enhances home safety while respecting the need for user privacy and autonomy. To do so, we have recommended data justice [38] as an equitable approach to these issues through regulatory guidelines.

Future directions for research in this area include studies on how privacy regulators can better support adults aged 50 years and older who use SHT or wearable devices for health or safety purposes. Further work is also needed on how privacy settings can be made more easily accessible and flexible to support everyday users in various contexts. As mentioned, robust analysis is needed where there is a current gap in the literature pertaining to the link between older adults, targeted advertisements or personalized insurance pricing, and SHTs or wearables [28], both in the practical application of such commercial relationships and through user perception studies. For further insight on this topic, subsequent user perception research on SHTs in general should actively include participants over the age of 50 years, especially in the oldest age categories, as opposed to targeting younger populations exclusively. Finally, beyond privacy and security, user perception studies on related ethical issues such as AI discrimination and the potential impacts on user autonomy should be further explored.

## Appendix

Multimedia Appendix 1 Search strategy.

Multimedia Appendix 2 PRIMSA Checklist.

## Abbreviations

AI: artificial intelligence
EbD: ethical by-design
PRISMA: Preferred Reporting Items for Systematic Reviews and Meta-Analyses
SHT: smart home technology
