# Supplementary material for: User Perception of Smart Home Surveillance Among Adults Aged 50 Years and Older: Scoping Review
Source: JMIR Mhealth Uhealth. 2024 Feb 9;12:e48526. doi: 10.2196/48526 (PMC10891486; doi:10.2196/48526)
Supplement: Multimedia Appendix 1 [file mhealth_v12i1e48526_app1.docx]

Search terms: (smart home*) OR (intelligent home*) OR (smart house*) OR (home automation) OR domotic OR smarthome OR homeseer OR homekit OR mihome OR homeOS OR “Google home” OR “Amazon home” OR (smart environment) OR “ubiquitous home*” OR (smart apartment) OR (home intelligence) OR (home network) OR (remote control home) OR (smart appliance) OR (ambient sensor) OR (Ambient assisted living) OR (Smart speaker) OR (Voice assistant) OR Agetech OR Gerontechnolog*) AND (surveill* OR security OR privacy OR (data collection) OR (data access) OR (data own) OR risk* OR benefit OR safety OR safe OR monitoring ) AND (focus group or qualitative or ethnographic or ethnography or fieldwork or "field work" or "key informant" OR interview OR interviews OR questionnaire OR questionnaires OR survey OR surveys OR themes OR phenomenology OR Delphi OR narration).
